# Supplementary material for: Occurrence of human infection with Salmonella Typhi in sub-Saharan Africa
Source: Sci Data. 2024 Oct 5;11:1089. doi: 10.1038/s41597-024-03912-x (PMC11455901; doi:10.1038/s41597-024-03912-x)
Supplement: Supplementary file 1 — Occurrence of human infection with Salmonella Typhi in sub-Saharan Africa [file 41597_2024_3912_MOESM1_ESM.docx]

# Supplementary Information

### Search string

1. PubMed

("Typhoid Fever"[Mesh] OR "Salmonella typhi"[Mesh] OR “typhoid,”[tw] OR “S. Typhi,” [tw] OR “Salmonella Typhi,” [tw] OR “enteric fever”[tw])

AND

hasabstract

AND

(Angola[tw] OR “Sub-Saharan Africa”[tw] OR “Subsaharan Africa”[tw] OR “Sub Saharan Africa”[tw] OR “Central Africa” [tw] OR Cameroon[tw] OR “Central African Republic” [tw] OR Chad[tw] OR Congo[tw] OR “Democratic Republic Congo” [tw] OR “Equatorial Guinea” [tw] OR Gabon[tw] OR “Sao Tome and Principe” [tw] OR “Eastern Africa” [tw] OR “East Africa” [tw] OR Burundi[tw] OR Djibouti[tw] OR Eritrea [tw] OR Ethiopia[tw] OR Kenya[tw] OR Rwanda[tw] OR Somalia[tw] OR “South Sudan” [tw] OR Sudan[tw] OR Tanzania[tw] OR Uganda[tw] OR “Southern Africa” [tw] OR “South Africa” [tw] OR Angola[tw] OR Botswana[tw] OR Lesotho[tw] OR Malawi[tw] OR Mozambique[tw] OR Namibia[tw] OR Swaziland[tw] OR Zambia[tw] OR Zimbabwe[tw] OR “Western Africa” [tw] OR “West Africa” [tw] OR Benin[tw] OR Burkina Faso[tw] OR “Cape Verde” [tw] OR “Cote d`Ivoire” [tw] OR Gambia[tw] OR Ghana[tw] OR Guinea[tw] OR “Guinea-Bissau” [tw] OR Liberia[tw] OR Mali[tw] OR Mauritania[tw] OR Niger[tw] OR Nigeria[tw] OR Senegal[tw] OR “Sierra Leone” [tw] OR Togo[tw] OR Comoros[tw] OR Mayotte[tw] OR Madagascar[tw] OR Sahel[tw])

1. Embase

(‘typhoid’:ti,ab OR ‘S. Typhi’:ti,ab OR ‘Salmonella Typhi’:ti,ab OR ‘enteric fever’:ti,ab)

AND

'Africa south of the Sahara'/exp OR ‘Sub-Saharan Africa’:ti,ab OR ‘Subsaharan Africa’:ti,ab OR ‘Sub Saharan Africa’:ti,ab OR 'Central Africa'/exp OR 'Central Africa':ti,ab OR 'Cameroon'/exp OR 'Cameroon':ti,ab OR 'Central African Republic'/exp OR 'Central African Republic':ti,ab OR 'Chad'/exp OR 'Chad':ti,ab OR 'Congo'/exp OR 'Congo':ti,ab OR 'Democratic Republic Congo'/exp OR 'Democratic Republic Congo':ti,ab OR 'Equatorial Guinea'/exp OR 'Equatorial Guinea':ti,ab OR 'Gabon'/exp OR 'Gabon':ti,ab OR 'Sao Tome and Principe'/exp OR 'Sao Tome and Principe':ti,ab OR ‘Eastern Africa’:ti,ab OR ‘East Africa’:ti,ab OR 'Burundi'/exp OR 'Burundi':ti,ab OR 'Djibouti'/exp OR 'Djibouti':ti,ab OR 'Eritrea'/exp OR 'Eritrea':ti,ab OR 'Ethiopia'/exp OR 'Ethiopia':ti,ab OR 'Kenya'/exp OR 'Kenya':ti,ab OR 'Rwanda'/exp OR 'Rwanda':ti,ab OR 'Somalia'/exp OR 'Somalia':ti,ab OR 'South Sudan'/exp OR 'South Sudan':ti,ab OR 'Sudan'/exp OR 'Sudan':ti,ab OR 'Tanzania'/exp OR 'Tanzania':ti,ab OR 'Uganda'/exp OR 'Uganda':ti,ab OR ‘Southern Africa’:ti,ab OR ‘South Africa’:ti,ab OR 'Angola'/exp OR 'Angola':ti,ab OR 'Botswana'/exp OR 'Botswana':ti,ab OR 'Lesotho'/exp OR 'Lesotho':ti,ab OR 'Malawi'/exp OR 'Malawi':ti,ab OR 'Mozambique'/exp OR 'Mozambique':ti,ab OR 'Namibia'/exp OR 'Namibia':ti,ab OR 'South Africa'/exp OR 'South Africa':ti,ab OR 'Swaziland'/exp OR 'Swaziland':ti,ab OR 'Zambia'/exp OR 'Zambia':ti,ab OR 'Zimbabwe'/exp OR 'Zimbabwe':ti,ab OR ‘Western Africa’:ti,ab OR ‘West Africa’:ti,ab OR 'Benin'/exp OR 'Benin':ti,ab OR 'Burkina Faso'/exp OR 'Burkina Faso':ti,ab OR 'Cape Verde'/exp OR 'Cape Verde':ti,ab OR 'Cote d`Ivoire'/exp OR 'Cote d`Ivoire':ti,ab OR 'Gambia'/exp OR 'Gambia':ti,ab OR 'Ghana'/exp OR 'Ghana':ti,ab OR 'Guinea'/exp OR 'Guinea':ti,ab OR 'Guinea-Bissau'/exp OR 'Guinea-Bissau':ti,ab OR 'Liberia'/exp OR 'Liberia':ti,ab OR 'Mali'/exp OR 'Mali':ti,ab OR 'Mauritania'/exp OR 'Mauritania':ti,ab OR 'Niger'/exp OR 'Niger':ti,ab OR 'Nigeria'/exp OR 'Nigeria':ti,ab OR 'Senegal'/exp OR 'Senegal':ti,ab OR 'Sierra Leone'/exp OR 'Sierra Leone':ti,ab OR 'Togo'/exp OR 'Togo':ti,ab OR 'Comoros'/exp OR 'Comoros':ti,ab OR 'Mayotte'/exp OR 'Mayotte':ti,ab OR 'Madagascar'/exp OR 'Madagascar':ti,ab OR 'Sahel'/exp OR 'Sahel':ti,ab

'Africa south of the Sahara'/exp OR ‘Sub-Saharan Africa’:ti,ab OR ‘Subsaharan Africa’:ti,ab OR ‘Sub Saharan Africa’:ti,ab OR 'Central Africa'/exp OR 'Central Africa':ti,ab OR 'Cameroon':ti,ab OR 'Central African Republic':ti,ab OR 'Chad':ti,ab OR 'Congo':ti,ab OR 'Democratic Republic Congo':ti,ab OR 'Equatorial Guinea':ti,ab OR 'Gabon':ti,ab OR 'Sao Tome and Principe':ti,ab OR ‘Eastern Africa’:ti,ab OR ‘East Africa’:ti,ab OR 'Burundi':ti,ab OR 'Djibouti':ti,ab OR 'Eritrea':ti,ab OR Ethiopia':ti,ab OR 'Kenya':ti,ab OR 'Rwanda':ti,ab OR 'Somalia':ti,ab OR 'South Sudan':ti,ab OR Sudan':ti,ab OR 'Tanzania':ti,ab OR 'Uganda':ti,ab OR ‘Southern Africa’:ti,ab OR ‘South Africa’:ti,ab OR 'Angola':ti,ab OR OR 'Botswana':ti,ab OR 'Lesotho':ti,ab OR 'Malawi':ti,ab OR 'Mozambique':ti,ab OR 'Namibia':ti,ab OR 'South Africa':ti,ab OR 'Swaziland'/exp OR 'Swaziland':ti,ab OR 'Zambia'/exp OR 'Zambia':ti,ab OR 'Zimbabwe'/exp OR 'Zimbabwe':ti,ab OR ‘Western Africa’:ti,ab OR ‘West Africa’:ti,ab OR 'Benin'/exp OR 'Benin':ti,ab OR 'Burkina Faso'/exp OR 'Burkina Faso':ti,ab OR 'Cape Verde'/exp OR 'Cape Verde':ti,ab OR 'Cote d`Ivoire'/exp OR 'Cote d`Ivoire':ti,ab OR 'Gambia'/exp OR 'Gambia':ti,ab OR 'Ghana'/exp OR 'Ghana':ti,ab OR 'Guinea'/exp OR 'Guinea':ti,ab OR 'Guinea-Bissau'/exp OR 'Guinea-Bissau':ti,ab OR 'Liberia'/exp OR 'Liberia':ti,ab OR 'Mali'/exp OR 'Mali':ti,ab OR 'Mauritania'/exp OR 'Mauritania':ti,ab OR 'Niger'/exp OR 'Niger':ti,ab OR 'Nigeria'/exp OR 'Nigeria':ti,ab OR 'Senegal'/exp OR 'Senegal':ti,ab OR 'Sierra Leone'/exp OR 'Sierra Leone':ti,ab OR 'Togo'/exp OR 'Togo':ti,ab OR 'Comoros'/exp OR 'Comoros':ti,ab OR 'Mayotte'/exp OR 'Mayotte':ti,ab OR 'Madagascar'/exp OR 'Madagascar':ti,ab OR 'Sahel'/exp OR 'Sahel':ti,ab
